# Exploring adolescence as a key life history stage in bioarchaeology

**DOI:** 10.1002/ajpa.24615

**Published:** 2022-09-09

**Authors:** Mary E. Lewis

**Affiliations:** ^1^ Department of Archaeology University of Reading Reading UK

**Keywords:** growth spurt, life history theory, paleopathology, puberty, sexual maturation

## Abstract

Adolescence is a unique period in the life history of an individual. It is characterized by a myriad of changes that bioarchaeologists are only just coming to appreciate, related to sexual maturation, linear growth, immunological transformation, and emotional and cognitive development. New methods allow us to measure this age of transition through the stages of the adolescent growth, as a proxy for the physical development associated with sexual maturation (puberty). This review outlines ways bioarchaeologists may draw on research developments from the fields of human biology, evolutionary theory and neurobiology to advance a more holistic approach to the study of adolescence in the past. It considers current theoretical and analytical approaches to highlight the research potential of this critical stage of life history. This synthesis integrates the most recent research in the medical sciences concerned with body and brain development, and outlines the biological processes involved with sexual and physical maturation of the adolescent. The goal of this review is to help inform potentially rewarding areas of research that bioarchaeologists can contribute to and draw from, as well as the challenges and limitations, theoretical and methodological questions, and ways in which we can develop the study of adolescence in the discipline going forward.

Adolescence has been a central concern to biologists, behavioral scientists, neurobiologists, geneticists, social historians, and health policy advisors for decades, but is only just emerging as an area of serious interest in bioarcheology. Adolescence is defined as the physiological and psychological transition from childhood to adulthood, which begins with invisible hormonal changes signaling the onset of puberty (Ellison et al., 2012). It is a complex and dynamic period that is contextually and individually tailored, with maturation of the body and brain intimately linked to social status, location, culture, environment, and family circumstances (Dorn & Susman, 2019). Puberty is a short‐term biological event taking place over a few weeks (Bogin et al., 2018), although it is associated with a longer period of physiological changes ending in sexual maturation, and incorporating complex and interrelated hormonal processes, and the adolescent growth spurt (Ellison et al., 2012). This pivotal developmental stage providing a critical window into genetics, prenatal stressors, and health, which has consequences across the generations (Dorn et al., 2019).

Given the increase in focus on the skeletal remains of children, absence of a discourse on adolescence is striking and ignores fresh opportunities for measuring and understanding health both today and in the past (Lewis, 2016). It is a fruitful area of study that bioarchaeologists are equipped to explore, and recent methods to allow the stages of the adolescent growth spurt to be mapped as a proxy for sexual maturation has allowed for new avenues of research. For example, adolescents differ in their psychological responses to puberty, and in the ages at which it begins (onset), when maturation milestones are reached (pubertal timing), the pace of development (tempo), and in the alignment of the different maturational changes with each other (synchrony) (Mendle et al., 2019). These features can all be measured in past populations using growth profiles and detailing skeletal maturation. Adolescents undergo changes to their immune system and brain function, that leaves them vulnerable to long term stress and can reactivate latent diseases, or cause the development of new ones (Dahl, 2004). Suboptimal decision‐making leaves adolescents susceptible to health insults due to addiction (alcohol, drugs), sexual activity and trauma as the result of risky behaviors and heightened male aggression (Brener et al., 2004; Peper & Dahl, 2013). In sum, adolescents provide many possibilities for pal‐eopathological research. They are a dynamic subgroup of any population. Adolescents are highly mobile, with 15–19 years being a key age for short and long‐distance migration (Erulkar et al., 2006; Scofield, 1971), an age at which many start adult work. They are often socially visible, with their actions documented in historical sources (Lewis et al., 2016). Adolescents may also become parents. Young mothers (under 20) are the most at risk of pregnancy hazards, with underdeveloped pelvises coupled with a propensity to make poor dietary choices, increasing the chances of neural tube defects and anemia in their offspring (Expert Consultative Group for Every Woman Every Child on Adolescent Health, 2015). All of this information is pertinent to our interests in the mother‐fetus nexus, frailty, energy trade‐offs, life history theory, adaptive plasticity, and the developmental origins of health and disease (DOHaD).

## 1 DEFINING THE “ADOLESCENT”

**FIGURE 1:**
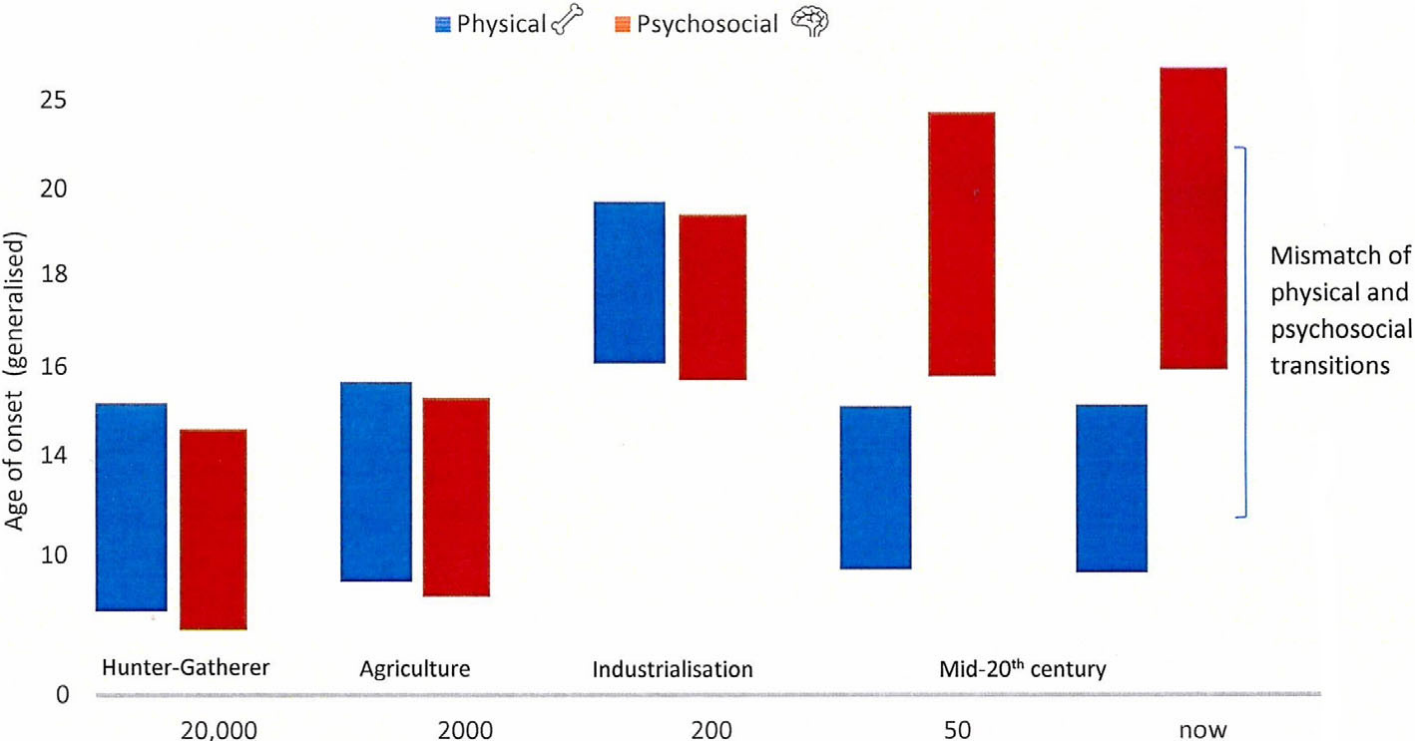
Relative mismatch of physical maturity (menarcheal age) and psychosocial maturity over time with increasing social complexity (adapted from Gluckman & Hanson, 2006: 11).

It is rare for older children to die and enter the archeological record, having survived the often hazardous events of birth and weaning. Hence, numbers of adolescent skeletons (traditionally 13–17 years) tend to be small, limiting meaningful research into this critical cohort. This can be resolved using the World Health Organisation (1993) standard ages for adolescents (10–19 years) or a vulnerable “young person” (10–24 years). Exploring adolescence in the archaeological record requires a biological age to be established, but with an understanding of the wider cultural context. Different societies may have had a very different concept of this transitional period. Our consideration of adolescence in bioarcheology needs to go beyond the physical to include cognitive development. The adolescent brain is dominated by the limbic system that controls impulses of pleasure and reward, this matures in advance of the prefrontal cortex that governs reasoning and timing, which is only fully developed around 25 years (Goddings et al., 2019). This psychological transition to adulthood is varied and culturally embedded (Coleman, 2011). Life history psychologists have argued that the mismatch between this emotional maturity and sexual development widens as human societies became more complex (Figure 1), increasing the need for an extended period of adolescence (Worthman et al., 2019). Something that archeologists are just beginning to explore (French & Nowell, 2022; Nowell & French, 2020; Nowell & White, 2010). This protracted brain development results in a longer period of heightened sensitivity to stress (Dorn et al., 2019; Tottenham & Galván, 2016), and suggests adolescence may also be a peak time for the development of certain skeletal and dental indicators of stress.

For biologists and psychologists, adolescence is broken down into defined age groups that could be useful in bioarcheology. Early adolescence is defined as the period between 10 and 14 years, mid‐adolescence 15–17 years, and late adolescence between 18 and 25 years (Barrett, 1996). Individuals within this latter category are also often referred to as “emerging adults” (Tanner & Arnett, 2016). These phases are associated with developing physical awareness and an incipient social identity, providing a potential model for exploring embodiment and agency (Table 1). We must be wary of transposing modern concerns about this age group onto the past. For example, it is likely that physical changes associated with sexual maturation were delayed into the middle adolescence in the postmedieval period (Diers, 1974). In other past cultures, the transition into an adult life was probably much shorter or less dramatic than it is today. Mead (1928) criticized Hall's (1904) argument that 12–25 years was period of universal and inevitable upheaval or, “storm and stress.” Mead had not seen any anxiety in the youth of Samoa who, from an early age were exposed to all aspects of adult life, including sexual relationships, which she argued eased the step into adulthood (Lerner & Steinberg, 2004). For women in medieval England, their status as adults often began with marriage, which for the nobility may have been as young as 12, while in the general population marriage occurred around 20–25 years (Shapland et al., 2015). Hence, social “adolescence” may have been long or short depending on the individual's culture, economic status, or gender.

**TABLE 1:** Physical and psychosocial characteristics associated with different ages (phases) of adolescence

Phase	Age	Physical characteristics	Psychosocial characteristics
Early	10–14	Rapid physical growth and sexual maturation Uneven growth results in awkward appearance Often tired	Worries about being normal Shyness, blushing, modesty Emerging sexual feelings Social contact with opposite sex often in groups
Middle	15–17	Continued physical development Excessive activity alternating with extreme lethargy Increase appetite Increased need for sleep	Adapt sexually and establish sexual identity Personal sense of masculinity or femininity Learn psychosocial rules surrounding sexual behavior
Late	18–24	Physical and sexual maturation mostly complete Greater acceptance of physical appearance	New sense of physical self Defined sense of identity Developed stable and productive relationships Meet demands of increasing roles and responsibilities

*Source*: Adapted from SAHRC (nd).

Fully exploring this life history stage relies on our ability to accurately estimate a physiological age to assign a chronological age. While this may not be an issue in the early phases when dental and skeletal development is at its peak, by the time an individual reaches middle and late adolescence the dentition is nearing completion, and few skeletal indicators remain. Once dental development is complete (at the end of the middle adolescent stage), development of the spine and pelvis becomes the focus. Lewis et al. (2016) assessed the development of the ischio‐pubic ramus (18–21 years), fusion of the ischial epiphysis (18–13 years), the first to the second sacral vertebra (closes 20–25 years); appearance of the epiphyseal flake for the medial clavicle (18–21 years) and vertebral annular rings (complete 18–23 years) to assign individuals to the late adolescent stage (Albert & Maples, 1995; Albert & McCallister, 2004; Cunningham et al., 2016). Any skeleton with a fused S1–S2 junction and fused medial clavicle epiphysis were considered adults (over 25). It is well understood that differences between age assigned using skeletal maturation and chronological age occur due to environmental and genetic factors including nutritional status, infections, low social status, and high altitude (Nahhas et al., 2013), but they served to divide the young adults into a more defined group, and allow an end point of maturation to be identified.

Males and females enter and progress through puberty at different ages, and so it is desirable to be able to estimate the sex of the adolescent based on skeletal morphological characteristics. In this case, sex is treated as binary only as a convenience rather than being conceived as a reality (DuBois & Shattuck‐Heidorn, 2021). Sex determination in nonadults is widely recognized to be problematic. Nevertheless, research on known‐sex child collections has proven fruitful. Several areas of the skeleton have shown to be more accurate with increasing age, with the best results reported for the ilium (Wilson et al., 2015). It is possible to provide a sex assessment, even if tentative, for nonadults from as young as 10 years based on features of the pelvis, mandible, and humerus, although it is recommended a sex is assigned only when the majority of traits, and all skeletal areas agree (Lewis et al., 2016). Reported accuracies for predicting sex vary depending on the population studied, with the most promising being the sciatic notch angle of the ilium (72%), the sciatic notch depth (81%), auricular elevation (72%–85%), humeral trochlear symmetry (81.5%), humeral olecranon fossa shape (85%), and the medial epicondyle angle (78%) (Falys et al., 2005; Rogers, 2009; Sutter, 2003; Wilson et al., 2015). While many cranial features will still be unpronounced in young males (Walker et al., 1988) risking a false female estimate, the mandible has proved to be more sexually dimorphic at a younger age, with chin prominence achieving 73% accuracy (Sutter, 2003). After 15 years (middle adolescence) or once the pelvis has fused, methods used to estimate sex in adults can be applied, avoiding the cranium (see Buikstra & Ubelaker, 1994). Future studies will benefit from new methods analyzing sex‐specific amino acid sequences from peptides in dental enamel (Parker et al., 2019; Stewart et al., 2017).

## 2 SKELETAL MATURATION AND THE GROWTH SPURT

The adolescent growth spurt is defined by skeletal growth as well as changes in the size and composition of multiple systems of the body (muscle, fat, metabolism). During this time, individuals gain 50% of their peak bone mass and adult body weight (Rogol et al., 2002; Tanner, 1962). The heart doubles in size and weight, growth of the lungs accelerates, and individuals increase their strength and endurance (Tanner, 1962). This makes the adolescent growth period a critical and sensitive stage in the healthy development of the individual.

**FIGURE 2:**
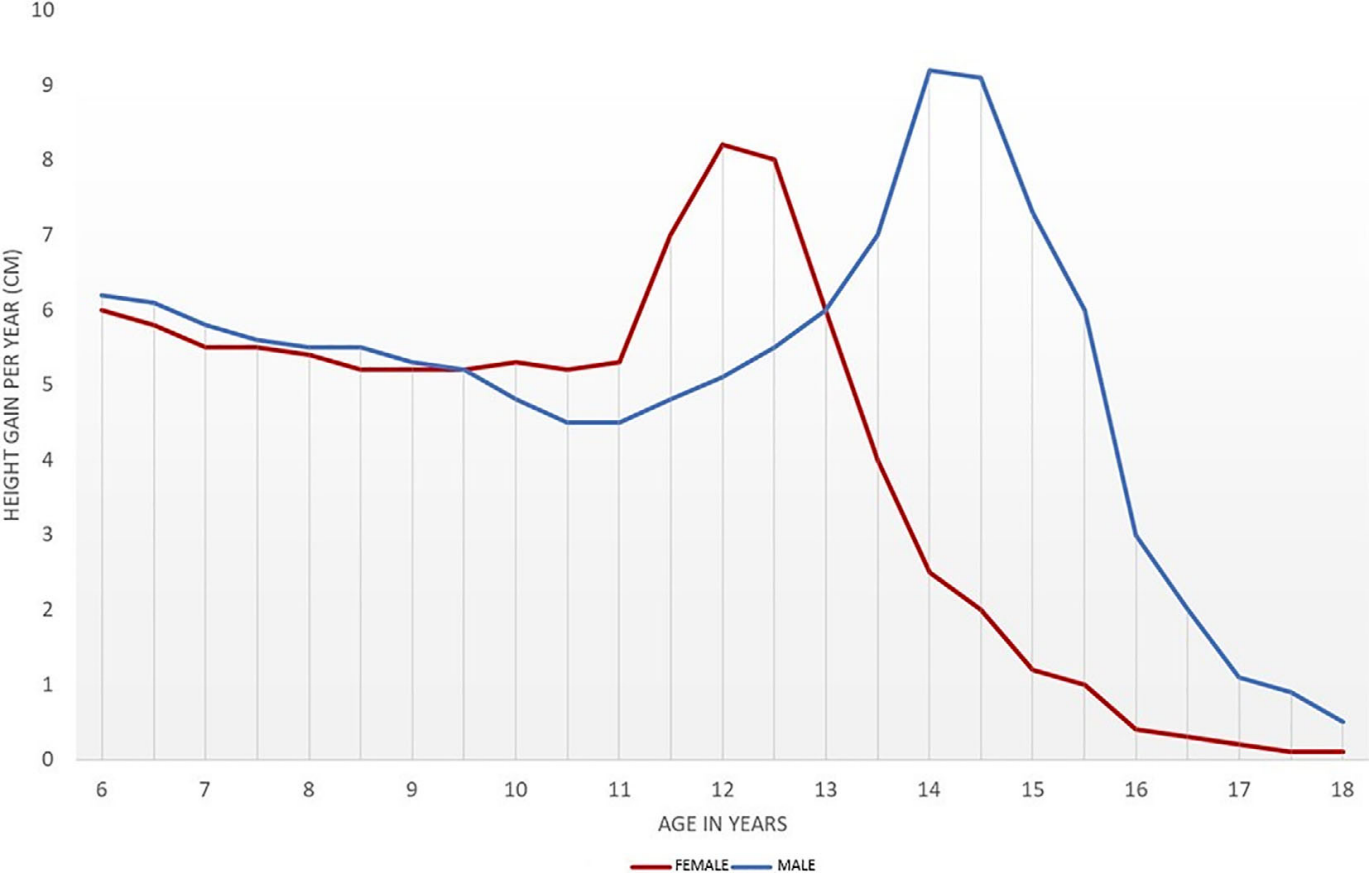
Pattern of adolescent growth for males and females. Curves are based on a fictional population with PHV between 12 and 13 years for females and 14–15 years for males.

This elevated period of growth is superseded by a slow growth period (SGP), or dip in the pace of growth (SGP), caused by a decrease in the levels of growth hormone (GH) released by the pituitary gland (Rogol et al., 2002). The adolescent growth spurt begins in earnest with the release of sex hormones by the hypothalamus and surging levels of the GH and insulin‐like growth factors (IGF‐I) (Mason et al., 2011). This significant period of growth can be simplified into a series of stages: acceleration, peak height velocity (PHV), deceleration, and completion (Figure 2). In the first 2 years of the growth spurt, linear growth is achieved through accelerated long bone length, but later is dominated by increasing trunk (spinal) height (Hornberger, 2005). The pinnacle of this growth spurt (PHV) occurs 1–2 years earlier in females than males, although the overall duration of the growth spurt is the same for both sexes. Today, PHV is reached between the ages of 11.6–12.5 years and 13.4–14.1 years in females and males, respectively (Malina et al., 2021). In males, PHV coincides with increased muscle mass and physical strength (Rogol et al., 2002), as well as attainment of an adult voice. In females, PHV coincides with breast development and other external signs of sexual maturation (Tanner, 1962). The dual action of the hypothalamus and pituitary gland (HPG‐axis) during physical growth and maturation is moderated by leptin and ghrelin. Leptin signals when there is adequate nutritional intake for the growth spurt, sexual maturation, and menarche (Kaplowitz, 2008; Yermachenko & Dvornyk, 2014). Ghrelin, or the “hunger hormone” is inversely correlated with the gonadal axis and delays sexual maturation in underfed children with insufficient energy reserves (Aslam, 2020). Hence, a delay in the onset of PHV (or tempo) in a skeletal sample may indicate poor nutrition in the individual or adolescent group under study.

As growth returns to normal levels (deceleration), women achieve menarche. Shortly after, the brain releases neuro‐endocrine signals in a 26‐day cycle (Zacharias & Wurtman, 1969; Zhang, Liu, et al., 2008; Zhang, Pollack, et al., 2008). It can take between 3 and 5 years for these menstrual cycles to stabilize and become regular (Mendle et al., 2019). Elevated estrogen levels stimulate endosteal bone apposition in females, and suppress subperiosteal bone apposition, while in males, lower levels of estrogen continue to promote both endosteal and subperiosteal bone apposition (Bass et al., 2002; Šešelj et al., 2012). The adolescent growth spurt comes to an end when chondrocytes in the resting zone of the growth plate reach the end of their proliferative capacity (senescence), and the invading bone cells result in epiphyseal fusion (Nilsson & Baron, 2004). This process is triggered by a rise in estrogen in females and estrogen receptors in the growth plate for both sexes, (Cutler, 1997; Nilsson et al., 2003). The pace and intensity of growth and accompanying sexual maturation, including menarche, is influenced by many varied and overlapping factors, including the presence of genetic and endocrine disorders, ancestry, social status, under or malnutrition, exposure to pollutants, extreme physical exercise, psychological stress, increased or reduced body mass, and chronic illness (Baxter‐Jones et al., 1994; Gluckman & Hanson, 2006; Goyal et al., 2012; Karapanou & Papadimitriou, 2010; Louis et al., 2008; Rosen & Foster, 2001; Tonstad & Sivertsen, 1997; Zacharias & Wurtman, 1969). Improvements in global nutrition and health have resulted in females achieving their final height around the age of 16 year compared with 18 or 19 years in the 19th century. Some males in 1880 did not reach full adult height until 25 years, compared with an average of 18 years today (Muuss, 1970).

Numerous growth studies in bioarcheology demonstrate a divergence in growth profiles from 10 years onward, whether expressed as diaphyseal lengths, or a percentage of attained adult height in that sample (Lewis, 2002). Several authors have explored these patterns in terms of the adolescent growth spurt (Lewis, 2002; Pfeiffer & Harrington, 2011; Piontek et al., 2001; Šereikine & Jankaukas, 2002). Numbers of adolescents in these studies are often small, and individuals in early studies often lack a sex estimation meaning the divergence may be an artifact of the different proportions of males and females contributing to these profiles (Lewis, 2002; Newman & Gowland, 2017). While a natural divergence may occur as females enter the growth spurt earlier than males, it is also true that any environmental hardships will have a greater impact on growth at this stage, due to the greater energy requirements of the spurt, contributing to shorter and perhaps stunted individuals in the mortality sample if conditions did not allow for catch‐up growth. Geber (2014) noted fewer growth arrest (Harris) lines in the 13–17‐year‐olds from his Irish famine sample, arguing that it indicated that their adolescent growth spurt had not initiated (and hence was not slowed causing a line), due to severe nutritional deficiency.

A pattern of extended adolescent (catch‐up) growth was identified by Cardoso and Garcia (2009) who found growth before puberty was delayed in both their medieval and early 20th‐century Portuguese samples, but postpubertal growth for the medieval population showed significant recovery with femoral lengths finally surpassing that of the 20th century group. Differences in the pace and timing of growth in different segments of the skeleton during adolescence has also been explored, especially in regard to the femur, humerus (Nowak‐Szczepanska & Koziel, 2016; Smith & Buschang, 2005), and vertebral column (Newman & Gowland, 2015). Newman and Gowland (2015) found that far from being complete at around 5 years, the transverse diameter of the vertebral neural canal in postmedieval individuals continued to increase into late adolescence, providing a new avenue to explore the timing of stress (Newman & Gowland, 2015). More recently, Dewitte and Lewis (2021) argued that reduced stature of London females after the Black Death demonstrated improved health after the pandemic, resulting earlier menarche and an earlier end to linear growth as an energy trade‐off response.

As a crucial period for healthy development (Hochberg & Belsky, 2013; World Health Organisation, 1993), the adolescent growth spurt is marked by a series of skeletal maturation markers and changes in bone density that provide a natural avenue for bioarchaeological research (Frisancho et al., 1970; Lewis et al., 2016). Future potential lies in the study of the SGP as a critical point for transgenerational fetal programing in males, referred to as the Paternal Origins of Health and Disease (POHaD) (Bygren et al., 2001; Pembrey, 2002; Pembrey et al., 2006; Soubry, 2018). Poor nutrition and exposure to psychological trauma in this preadolescence period in males detrimentally impacted the health of their male offspring. It is not clear if the association of male‐line transgenerational changes are due to an epigenetic event that is sperm‐led, or a genetic evolution that is Y chromosome specific (Isganaitis et al., 2017; Ryan & Kuzawa, 2020; Whitelaw & Garrick, 2006). By understanding the growth spurt we can develop measures to track changes and explore factors that may impact the ability of adolescents to reach their full growth potential. It is acknowledged that the *pace* of growth is more important than attained height in understanding environmental stress (Bogin et al., 2018; Hermanussen, 2010). However, methods to measure the tempo of growth in cross‐sectional archeological data present more of a challenge.

## 3 PUBERTY STAGE ESTIMATION IN BIOARCHAEOLOGY

**FIGURE 3:**
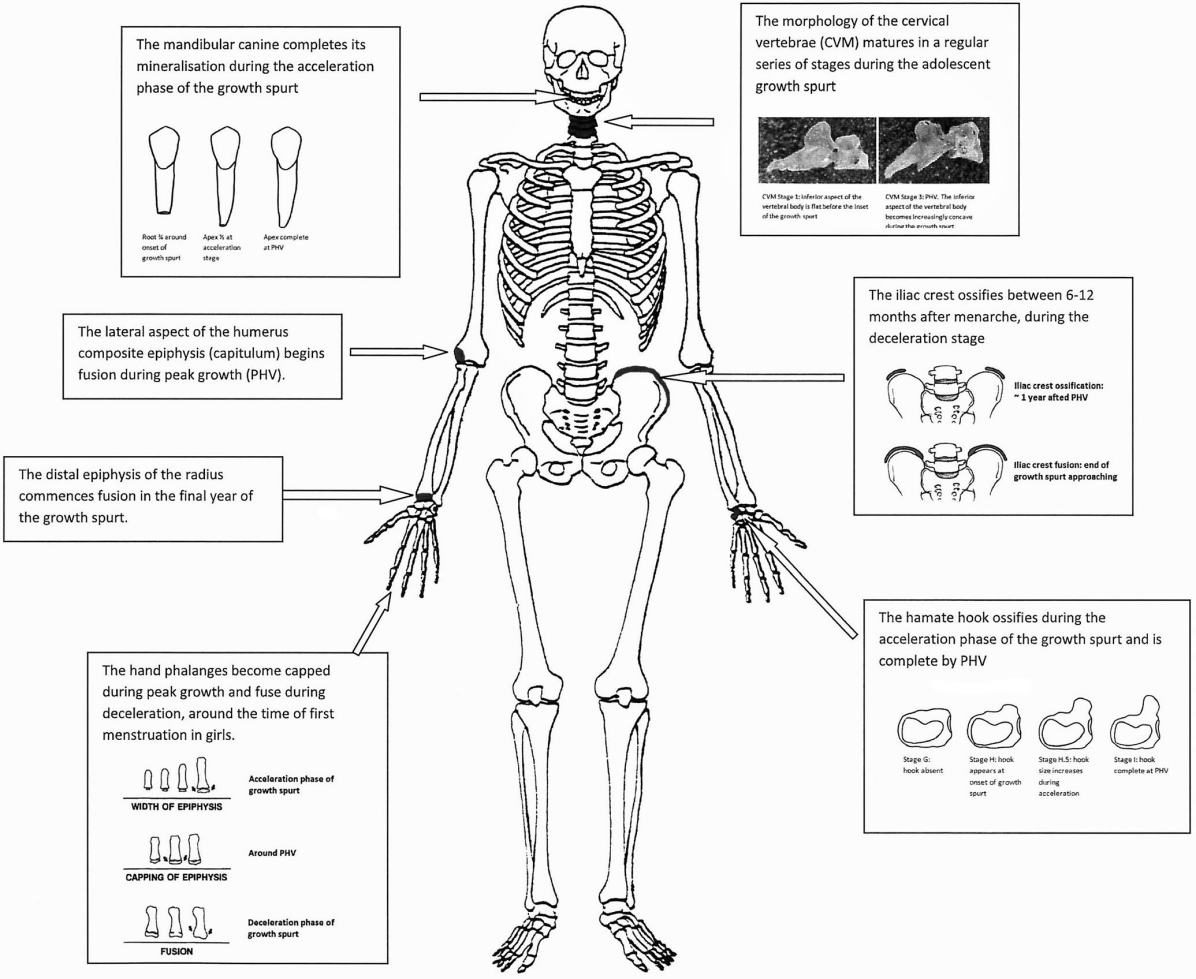
Location and characteristic of skeletal features used to trace puberty and the adolescent growth spurt in archeological remains (adapted from Shapland & Lewis, 2013).

Shapland and Lewis (Lewis et al., 2016; Shapland & Lewis, 2013, 2014) proposed a series of stages, from prepuberty to completion, that could be assessed using skeletal maturation and changes in morphology, to determine progress through the adolescent growth spurt, as a proxy for sexual maturation (Figure 3). This includes ways to identify the key life history stages of PHV and the average age of menarche.

**FIGURE 4:**
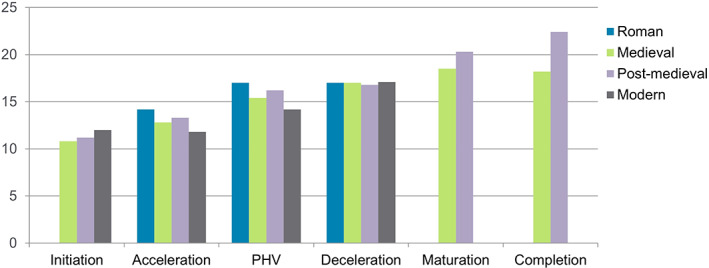
Average age of males in each stage in archaeologically derived adolescents from England compared with the age of entry to each stage in modern males.

**FIGURE 5:**
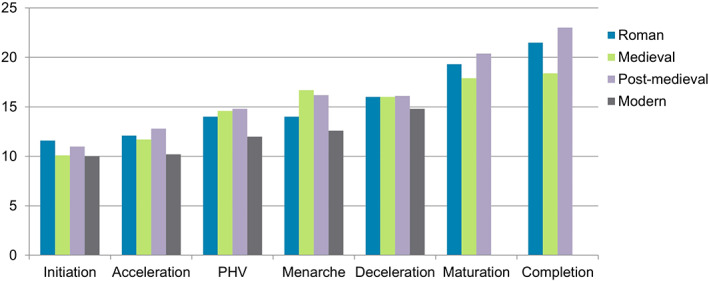
Average age of females within each stage in archeologically derived adolescents from England, compared with the age of entry to each stage in modern females.

These methods have been applied to individuals aged 10–25 years from different archeological contexts and periods (Doe, Molina Moreno, et al., 2019; Doe, Pérez, et al., 2019; Arthur et al., 2016; Henderson & Padez, 2017; McGovern, 2019; Valmé, 2019). Most data come from England, where studies have been carried out in Roman, medieval and postmedieval adolescent samples, with sex estimation (following Shapland & Lewis, 2013) of the most well‐preserved skeletons allowing for a more detailed overview. All of the adolescents show a similar age of onset (around 10 years), Roman males (based on small numbers) lag behind their medieval counterparts (Figure 4). Romano‐British females have a later age of onset but achieve menarche nearly 2 years earlier than the medieval women (Figure 5). Skeletal maturation continues into the 20s for postmedieval adolescents but is complete for most 18‐year‐olds in medieval England.

The employment of modern clinical markers to track the progress of puberty in archeological skeletal remains is not without its problems. Many modern studies use longitudinal data and growth velocity to calculate the onset and tempo of puberty in healthy living children of known age and sex. Archeological data is cross‐sectional and based on, often small numbers of children who died during adolescence, either through acute or chronic diseases, or fatal injury. We cannot assess velocity of growth and therefore rely on individual independent measures of canine development, epiphyseal fusion, and skeletal morphological changes to track the growth spurt. The markers that link sexual maturation with skeletal and dental development are derived from modern, mainly healthy children, and we cannot know if the same correlations between hormonal and physiological change existed in the past (Doe, Pérez, et al., 2019). Epiphyseal fusion of the radius and iliac crest that coincides with the deceleration phase of the growth spurt in modern practice, is based on radiographic assessments that may record “fusion” slightly earlier than it is observed in dry bone (Cardoso, 2008; Krogman & Iscan, 1986). Just as new imaging techniques may see a decline in the age at which ossification of the iliac crest (hence menarche) can be observed (Lottering et al., 2017). There is a danger in comparing archaeological with modern ages of attainment due to secular changes that will affect prediction of chronological age from dental and skeletal development. Dental age assessment in older adolescents relies on the third molar, a tooth that is notoriously variable in the correlation of age with formation and calcification stages. Individuals estimated to have a mean age of 17 years could be between 14 and 21 years (Liversidge & Marsden, 2010: 4), making direct comparisons of the timing and tempo of growth with modern populations difficult. In fact, evidence is mounting that the third molar is less reliable than skeletal maturation for estimating chronological age in the later stages of puberty (Cardoso et al., 2018). The increasing lag of archeologically derived adolescents as they progress through the stages of the growth spurt may be, in part, an artifact of a lag in mineralization of the dental roots in these older ages, which have been found to be delayed in comparison to modern children in conditions of poor nutrition (Cardoso et al., 2010).

The age of puberty onset (10–12 years) reported in archeological studies where both age and sex are estimated, is suspicious. Males should lag females by 1–2 years and there is a normal variation of 4–5 years in the timing of the onset of puberty in healthy humans (Parent et al., 2003). While archeological studies have identified early and late developers in each sample at each developmental stage, markers used to assess onset (canine root, hook of the hamate bone) may not be sufficiently sensitive to capture the true pattern of puberty onset. Finally, the ages used in modern assessments for puberty stages use ages “at entry” for each developmental stage, as compared with the mean ages of those “in the stage” in archeologically derived studies (Arthur et al., 2016), although providing ranges for the youngest and oldest children at each stage can illustrate whether the range of normal variability or delay is present (Table 2).

**TABLE 2:** Variations in the mean dental age (in years) of all individuals (males and females combined) in each pubertal stage in medieval England (Lewis et al., 2016)

Stage	Number	Mean age	Youngest	Oldest
Acceleration	159	12.4	9.9	16.6
PHV	127	15.1	11.2	19.3
Deceleration	197	16.2	12.5	19.5
Maturation	48	18.0	15.2	22+^a^
Completion	121	22+^a^	16.2	25^a^

^a^ Skeletal age.

Henderson and Padez (2017) noted slight asymmetry in epiphyseal fusion that may result in one side being assigned a different stage to the other. Taking a robust approach, requiring three or more traits to be observed before assigning a puberty stage considerably reduced the sample size from an initial 994 to a sexed and dentally aged sample of just 236 individuals (Lewis et al., 2016). The requirement for a dental age and complete skeletons means that sample sizes are often too small to form any conclusions. We should include individuals as young as 8 years to ensure the full range of variation for early and late onset is captured, and more research into known age and sex groups may help refine the details ‐ although these are likely to be biased toward larger postmedieval samples. The presence of early and late developers means we also need to include individuals up to 25 years (if not beyond) to cover the full range of the adolescent growth spurt and sexual maturation, and this will help to increase sample sizes.

The age of menarche has been a specific focus in historical and clinical literature as it provides a definitive stage of which to measure normal development and is affected by numerous stressors experienced at different life history stages. It forms the basis for research into fertility and life span for evolutionary anthropologists and in many societies is accompanied by a change in status for the female. The fragile nature of an unfused iliac crest means it often fails to survive in the burial environment, but it is the ossification of the iliac crest that is so clearly associated with the onset of menarche in females (Shapland & Lewis, 2013). Lacoste Jeanson et al. (2016) suggested the development of the premolars could be used to assign females to a pre‐ or post‐menarcheal group, but cautioned that the age of menarche and development of the premolars in their modern French sample may be coincidental, and that the method needed to be tested in known menarcheal status females from other countries and backgrounds, with different ages of menarche. There are no known “age at menarche” skeletal collections and so we rely on clinical records that, while they may provide views of the teeth, cervical vertebrae, and hand bones as standard practice, rarely include images of the elbow joint or pelvis.

Mortality bias may mean puberty attainment in the cemetery sample is not a reflection of the living sample from which they were derived. Puberty onset, menarche and the adolescent growth spurt are environmentally sensitive and stress experienced during later childhood and early adolescence can cause it to be delayed. These factors may also have led to frailty and death of the adolescents we are hoping to measure these events in. For example, the age of later medieval females outside London with a mean dental age, and an ossified but unfused iliac crest (around menarche) ranged from 16 to 17 years, whereas in London it was later at 17–19 years. While historical sources indicate this event usually occurred around the age of 12–15 years, suggesting a mortality bias (see Green, 2005). However, when Henderson and Padez (2017) tested the method on 55 known age and cause of death males and females from early modern Coimbra in Portugal. Females achieved menarche at 15 years, consistent with historical sources for Portugal at that time, with the youngest female in their sample achieving menarche at 12 years. It may be argued that comparing differences in the ages of development for archeologically derived adolescents against each other, rather than against modern data would reduce problems with mortality bias, as the level of errors in the mortality groups would be the same. However, recent research has indicated that the degree of hidden heterogeneity and hence mortality bias may vary between archeological samples and should be considered when trying to understand our data in the context of modern patterns (Spake, 2020; Spake & Cardoso, 2019). Many of the 10–25 year olds in our sample could also have died in accidents, that would not affect their normal development, due to their propensity for misadventure.

## 4 THE ADOLESCENT MOTHER‐FETUS NEXUS

The timing of puberty is a complex alchemy of fetal programing (or preconditioning), life experience, and genetics. This includes shorter‐term adaptation to severe stress, illness or undernutrition as the result of social‐economic status or accumulated childhood hardships. Puberty is not a newly established event; it is the result of a reawakening of gonatrophin‐releasing hormone (GnRH) network in the hypothalamus established prior to birth. As such, it is not surprising that fetal and perinatal experiences influence the onset and pace of sexual maturation (Aylwin et al., 2019; Gunnar, 2019). Epigenetic processes that cause long and short‐term modifications on genes help to explain how the timing and tempo of puberty, that is highly genetically controlled, can be altered by environmental and psychosocial forces (e.g., poverty, poor nutrition, exposure to alcoholism or tobacco smoke, physical neglect, death of a parent, war) (Boynton‐Jarrett & Harville, 2012; Dorn et al., 2019; Dossus et al., 2012; Karapanou & Papadimitriou, 2010; Wojtyla et al., 2012; Worthman et al., 2019). The fetus will predict the conditions it is likely to be born into based on the transfer of nutrients and hormones from the mother (nutritional homeostasis). This homeostasis is based on the mother's long term nutritional status and exposure to stress, buffering the fetus from short‐term fluctuations in environmental circumstances, known as “phenotypic inertia” (Kuzawa & Fried, 2017; Wells, 2010). Intergenerational exposure to adverse conditions by the parents, perhaps as the result of structural inequality on the basis of “race,” gender or any other related framework, program the fetus to expect a shorter life span and reduced reproductive capacity. This predictive adaptive response may result in earlier sexual maturation (e.g., menarche) to allow for earlier reproductive success (Hochberg & Belsky, 2013; Wojtyla et al., 2012). While this may be beneficial to ensure their survival, it can also cause problems if miscalculated (Low et al., 2012), with babies born into better than expected circumstances programed to store nutrients unnecessarily (Barker, 2012; Syme & Hagen, 2020; Wojtyla, 2011).

Most of the epigenetic traits or adjustments are “cleared” with each generation, but it has become increasingly apparent that some traits are carried across to subsequent generations (epigenetic inheritance) (Ryan & Kuzawa, 2020; Whitelaw & Garrick, 2006), with consequences for long term health and disease risks. DOHaD hypothesis has been explored in bioarchaeology in relation to the study of young children, bone plasticity and dental signs of rickets pertaining to the intimate mother‐infant nexus (Agarwal, 2016; Brickley et al., 2019; Gowland, 2015; Klaus, 2014; Temple, 2019). Puberty is another crucial stage in this generational life history approach and is reflected in the timing of menarche and influences future generations through the health of adolescent parents (Wojtyla et al., 2012). While early menarche may be a positive reflection of developmental plasticity in times of hardship, Ellis (2004) has argued that earlier menarche may risk the health of the offspring with higher levels of fetal wastage and reduced fetal growth.

Today, 85% of adolescent mothers are in the developing world, and 25% of all maternal deaths occur between the ages of 15–19 years (Conde‐Agudelo et al., 2005). In India, 40% of deaths of women aged 15–19 years occur during pregnancy and childbirth (Bhat, 2002). Under 16 years, mothers are more at risk of preterm birth, stillbirths, and neonatal deaths (Dickins et al., 2012). Young mothers and their babies are at risk primarily because of cultural or psychosocial factors. Smoking and drinking, economic instability, lack of breastfeeding, limited parental investment, or (today) dieting causing a mismatch between the womb and birth environment have all been cited as contributing factors (Torvie et al., 2015; Wojtyla, 2011). Well‐nourished and healthy adolescent females are seen as the key for overall population well‐being, with the nutritional levels of women before their first pregnancy having a direct impact on the development and birth‐weight of their child and its subsequent survival (Expert Consultative Group for Every Woman Every Child on Adolescent Health, 2015). The consequences of stress experienced by the mother prior to pregnancy, and its impact on maternal nutritional homeostasis and the developmental plasticity of the fetus highlight the intergenerational impact of social inequality on health (Kuzawa, 2020; Wells, 2010). Competition for nutrients between mothers who are still growing and the developing fetus can result in a small baby and a myriad of future health issues for the child (Gluckman & Hanson, 2006). The nutritional status of the mother at ovulation is also relevant during the period of embryogenesis when intensive process of DNA synthesis and oocyte development is at a susceptible phase, and can impact fetal growth (Jazwiec & Sloboda, 2019). The babies of young mothers are more likely to be of very low birth weight, (Torvie et al., 2015), with low birth weight in turn associated with early menarche, and a risk factor for developing adult‐onset diabetes (Sawyer et al., 2012; Whitelaw & Garrick, 2006).

Physical constraints also make young pregnancies risky. The pelvis continues to grow after the growth spurt has ceased, and if fecundity outpaces full development of the pelvis obstructive labor is a risk. The 2–5 year anovulatory period after menarche is argued to have been programed to reduce this risk (Gluckman & Hanson, 2006). If the average age of menarche was between 14 and 16 years in the past (Lewis et al., 2016), then women had the potential to give birth between 16 and 18 years of age. McGovern (2019) demonstrated a decline in the percentage of Romano‐British females with a narrow or contracted pelvis with age, from 50% between 14 and 16 years, to 18% by 20 years, with most of the pelvic growth occurring during the maturation phase, after menarche. Indeed, there are several examples of adolescent mothers who died in childbirth in the archeological record (Evison & Annable, 1994; Leeds & Harden, 1936; Lieverse et al., 2015; Malgosa et al., 2004) including a woman, possible aged as young as 16 years old from An Son on Van Co Don River in southern Vietnam, who died during a breech birth and with a pelvis that was still developing (Willis & Oxenham, 2011). These cases illustrate that middle and late phase adolescent women, at least from the prehistoric period, were fertile and having children.

## 5 A PALEOPATHOLOGY OF ADOLESCENCE

Adolescent health is receiving increasing attention in modern populations, with their unique behavior directly linked to morbidity. Their psychosocial development makes them more susceptible to accidents and suicides, and health risks as the result of smoking, alcohol, and sexual activity, but may be tempered by strong family of societal ties (Dahl, 2004; Viner, 2008). Adolescents represent a unique cohort in the epidemiology of disease as they sit at the crossroads between late onset childhood diseases and early onset adult diseases, and can suffer from both child and adult forms of cancer (Viner, 2008; Ward et al., 2014). In addition, the pubertal growth spurt, nutritional requirements, and immunological transition makes them susceptible to a tapestry of conditions. Some are unique to the adolescent, such as idiopathic scoliosis, early onset idiopathic arthritis, and mental disorders.

### 5.1 Trauma and mechanical stress

The age of menarche sets the scene for osteoporosis, with females who experience early menarche having less time to acquire subperiosteal bone and cross‐sectional bone strength, leaving them at increased risk of fractures and osteoporosis in later in life (Bonjour & Chevalley, 2014; Chevalley et al., 2009). Rapid skeletal growth, particularly of the lower legs and spine during puberty, mean adolescents are susceptible to conditions such as Scheuermann's disease, scoliosis (Burwell, 2003), slipped femoral epiphysis (Puylaert et al., 2004), spondylolysis and osteochondritis dissecans (OCD) (Kessler et al., 2014), with Osgood–Schlatter's disease and Schmorl's nodes also making an appearance in adolescence (Patton & Viner, 2007; Resnick & Goergen, 2002; Waldron, 2009). Froehle et al. (2017) reported that earlier menarche and subsequent lower stature resulted in more static and dynamic valgus knee movement, making females susceptible to greater knee injuries during strenuous activity and the development of arthritis in the longer term. The largest study of adolescent health based on skeletons from medieval England identified and increase in spinal and joint lesions (osteoarthritis, OCD, Schmorl's nodes) from the age of 14 years in both urban and rural samples, but almost double the levels of these conditions in the urban adolescents overall (20.5% urban; 9.4% rural). Although urban males had more OCD than females, the prevalence of OCD in the knee was over twice as prevalent (27.7%) in females than in the males (11.4%). Patterns of trauma also suggested urban males were engaged in interpersonal violence while the location of most lesions in the females suggested the impact manual labor was having on their knees and spines (Lewis, 2016).

### 5.2 Metabolic conditions

Adolescents have different nutritional requirements to the rest of the population, including increased need for iron, and vitamins A and D (Brabin & Brabin, 1992). Although some studies have shown iron deficiency has to be severe to affect growth (Ulijaszek, 2006), others argue that iron coupled with vitamin A deficiencies may slow the tempo of growth, with any catch‐up growth experienced later in pubertal development further depleting stores of these nutrients. Vitamin A, present in meat, poultry, fish and dairy products, fruit and vegetables are essential for healthy fertility (Brabin & Brabin, 1992). Increased demand for calcium and vitamin D during puberty can result in rickets and osteomalacia, and delay the growth spurt if they are not met (Moncrieff et al., 1973), and a link between vitamin D deficiency and early menarche in females from Bogotá, Colombia, has been suggested (Villamor et al., 2011). While this association has yet to be fully explained, it suggests a connection between vitamin D, sexual maturation and genetic variants in the estrogen gene (Chew & Harris, 2013). Adolescent females are particularly prone to low vitamin D blood serum levels compared with males, who were shown to have higher levels and greater vitamin D intake in a modern European study (Spiro & Buttriss, 2014). This difference may be the result to an early peak in the growth spurt in females that increases their requirement for vitamin D. Paleopathological research has identified angular deformities related to adolescent vitamin D deficiency in the sacrum, sternum, and knees (Lockau et al., 2019; Tschinkel & Gowland, 2020). These are among the last areas of the skeleton to fuse, making them prone to deformation before their morphology is locked in place. The relationship of sternal deformity with vitamin D deficiency was demonstrated by D'Ortenzio et al. (2016) who found a correlation of the deformity in adult individuals showing dental signs of rickets at 12.5 years.

### 5.3 Inflammatory diseases

Difference in immune system capability between males and females appear during adolescence as androgens and oestrogens reveal different moderating effects on the immune system. Testosterone is more immunosuppressive, while estrogen has been linked to suppressed cell‐mediated immunity, advanced B lymphocyte activity and antibody production (McDade, 2003). Hence following puberty, females are more prone to autoimmune diseases, and males more likely to experience chronic inflammatory disease and infections (Bupp, 2015). Hormone changes during each menstrual cycle exacerbate any underlining autoimmune conditions (Oertelt‐Prigione, 2012). Chronic bowel disorders such as Crohn's disease and colitis, often manifest in early adolescence and are associated with a delay in puberty development, particularly delaying PHV in males (Proos & Gustafsson, 2012), as the result of the influence of both poor nutrition and inflammation on the GH/IGF‐I axis (Mason et al., 2011). For example, 56% of children with Crohn's have growth delay. However, malnutrition is only thought to account for 60% of final growth impairment, with the direct effect of pro‐inflammatory cytokines on the growth plate accounting for the remaining 40% (Sederquist et al., 2014). Proinflammatory cytokines have a direct effect on the gonads and inhibit sex hormones by suppressing GnRH (Sederquist et al., 2014). An increased susceptibility to inflammation during adolescence is demonstrated by an increase in gingivitis, that peaks between 12 and 13 years (at the onset of puberty) because of the direct effect of steroid hormones on the periodontium (Mariotti & Mawhinney, 2013). The impact of inflammation on pubertal development is also seen in juvenile idiopathic arthritis that commonly manifests between 11‐ and 14‐years delaying menarche in affected females by up to 2 years compared with their healthy peers (Tsatsoulis et al., 1999; Umlawska & Prusek‐Dudkiewicz, 2010).

### 5.4 Chronic infection

Adolescents are increasingly susceptible to developing chronic infection such as tuberculosis and leprosy, due both to exposure to new diseases associated with a riskier lifestyle and the maturation of the immune system (Marais et al., 2005; Patil, 2013). With an increase in demand for resources during the pubertal growth spurt, the trade‐off between the immune system and physiological development becomes more apparent with mortality from infection increasing to 2.5 times that of late childhood (McDade, 2003). The greater propensity for adolescents to develop chronic tuberculosis for instance, is explained by Marais et al. (2005). They point to the destructive containment of pathogens characteristic of a maturing immune response potentially producing an oxygen rich environment which allows *Mycobacterium tuberculosis* to flourish, causing adult‐type cavitations in the lung tissue. The influence of the sex hormones on cellular and hormonal immune response may explain why at puberty, adolescents develop adult‐type tuberculosis, where bacilli are no longer contained but attacked, increasing the risk of the mycobacteria being released into the bloodstream (Marais et al., 2005; Patil, 2013). Mansukoski and Sparacello (2018) compared the cross‐sectional area of long bones in adolescent (18–25 years) and adult TB and non‐TB sufferers from 19th to 20th century Finland. They found tuberculosis sufferers had smaller cross‐sections in the femur and particularly in the humerus than the nontuberculous individuals. They argued this may have been the result of reduced subperiosteal bone development during adolescence as the result of infection, co‐morbidity with vitamin D deficiency or immobility. A Neolithic adolescent (14–16 years) with TB also showed more gracile bones compared with the rest of the adolescent group (Sparacello et al., 2016). Studies of males with pulmonary TB have demonstrated the impact of cytokines released during the disease on the endocrine system and on the release of adrenal steroids (Bozza et al., 2007). Those with an active infection had decreased levels of testosterone, but significantly elevated levels of GH and IGF‐I and inhibition of DHEA by the adrenal gland (del Rey et al., 2007) indicating that we might expect higher growth velocity but retarded development of secondary sexual characteristics. The complex link between TB and malnutrition might also inhibit this extra growth capacity (Cegielski & McMurray, 2004). The impact of TB on menstruation was identified in a study by Hassan and Darwish (2010), where 66% of women with pulmonary TB reported amenorrhea (26.5%), hypomenorrhea (20%), and fertility complications. The relationship between tuberculosis and puberty has also been noted in medieval individuals from England. Lewis et al. (2015) recorded a significant delay in progress through the puberty stages with tuberculosis, with double the cases in females. However, numbers were too small to link TB specifically with a delay in reaching deceleration (and hence menarche).

### 5.5 Cancers and pollutants

Early menarche puts females at greater risk of breast, ovarian, and endometrial cancers (Day et al., 2017). A lower age of puberty onset in males makes them more susceptible to testicular cancer in later life (Golub et al., 2008), perhaps due to their extended exposure to the sex hormones that act as growth factors in reproductive cancers (Susman & Dorn, 2009). When studying adolescents from urbanized societies, high exposure to lead (3 mg/dL or 0.144 mmol/L blood levels) has been shown to delay physical growth, maturation and menarche, perhaps due to its effects on endocrine system (Selevan et al., 2003; Ulijaszek, 2006; Wu et al., 2003). By contrast, exposure to mercury is thought to stimulate early menarche, when compared with those not exposed (Denham et al., 2005). All factors that we could consider when studying adolescents exposed to pollution in the past.

## 6 UNTAPPED POTENTIAL: THE FUTURE OF ADOLESCENT BIOARCHEOLOGY

Adolescence is a unique period of our development. The onset of puberty, commencement of the growth spurt, and the age of menarche are the result of a complex interaction between genetic signaling, fetal programing and shorter‐term adaptation to severe physiological and psychological stress. Adolescents provide a vehicle from which we can contribute to discourses on the mother‐fetus nexus, physiological stress, energy trade‐offs, life history theory, and the epigenetic origins of health and disease (DOHaD and POHaD). New methods allow us to measure the age of transition through various stages of the growth spurt as a proxy for the physical development associated with sexual maturation. The timing, tempo and synchronicity of these changes offer the opportunity for us to explore health in the past in greater detail. Comparisons of adolescents from different periods, cultures, social status, and heritage can provide an insight into different levels of stress, illness or undernutrition. Incremental analysis of isotopes within the dentine of adolescents (or dentine forming during this period) offers future avenues for exploring their diet in relation to health and identity (Avery et al., 2021). While often considered beyond the reach of bioarcheology, psychosocial stress has a direct impact on the age of puberty onset and menarche, and calls for a greater recognition in the past. For example, father absence was common in postmedieval England (Horrell et al., 1998) and we have access to the skeletal remains of individuals who lived through wars and famines, that offer an opportunity to explore the impact of these stressors on offspring, and perhaps transgenerationally. With industrialization, more young people would have traveled to large urban centers, causing not only health and psychological issues but also potential exposure to violence and pollution, and new habits such as smoking and drinking.

At what age the transition to adulthood occurred in each society in the past is culturally specific and may not have been based purely on physical development. It may be only understood by referring to the burial treatment of different age cohorts, or where they exist, historical records (Lewis, 2019). Paleopathological evidence signaling greater physical stress may indicate the age at which full adult labor was expected. Evidence for combat trauma or their presence in war graves would indicate when they were called on to fight. Hence, our understanding of adolescence needs to be understood within a cultural context. Perry (2006) cautioned that in the Byzantine Near East, marriage and childbearing occurred around 13–15 years. At this age women were entering a period of self‐sufficiency, and increased risks associated with childbirth. However, “those strictly adhering to western biological age categories…would identify these married, independent adults as ‘subadults’” (Perry, 2006: 97). In other groups, the age at which women reached sexual maturity and even marriage was still considered too young, and too risky for childbirth (Stoertz, 2001). We now have the opportunity to explore the reaction of different societies to puberty and adolescence, and any mismatch between sexual maturity and psychological behaviors in increasingly complex societies through time, that have to date only been hypothesized.

Closer collaboration with historians would allow us to develop a richer understanding of the factors that may have affected adolescents, and society's attitudes toward them. Even at an individual level, puberty assessment provides a detailed, contextual osteobiography (Hosek & Robb, 2019), allowing us to discern the identity of an adolescent who died with the physical appearance of a child (i.e., before PHV) or an adult (at deceleration), and through their burial, the different value they may have had in that community (Fricke et al., 2020).

We are well‐equipped to explore this age group, the growth spurt not only signals an increase in diaphyseal lengths but also bone mineral density, changes in the shape of the pelvis and dimensions of the spine. Any stunting or delay in development provides more precise age markers by which to track periods of stress. New proteomic methods for sex determination mean that tracing early pubertal development between the sexes is within our reach, and in turn, can help us elucidate growth profiles. There is a need for methodological refinement for identifying the age of menarche, exploring synchronicity of maturational factors and asymmetry, and selective mortality and hidden heterogeneity when comparing age at menarche from historical and archeological records. New approaches, such grouping adolescent females into pre‐ and post‐menarche groups (e.g., before and after deceleration) allows for greater sample sizes and does not require the precise age of menarche to be estimated (DeWitte & Lewis, 2021).

The health of adolescents not only reflects their own experience, or that of their parents and grandparents, but also that of their own offspring making it important crossroads in human health. We can explore inflammatory effects on skeletal maturation through evidence of adolescent periodontal disease and TB. In addition, developmental stress or trauma that occurs during adolescence (e.g., enamel hypoplasia on the third molar; Harris lines on the distal radius, femur and tibia, delayed spinal development, deformities of the sternum and sacrum) can be traced in older adults to provide an indirect exploration of adolescence. The presence of severe spinal lesions and arthritis in individuals as young as 20 years in medieval England suggest that “old age” based on skeletal degeneration needs to be reconsidered. Hard physical labor at a time when the spine and joints are so vulnerable, will cause skeletal aging to be accelerated (Lewis, 2019).

While the potential for the bioarcheology of adolescence is exciting, there remain challenges, and we must be cautious when comparing the data of our adolescent nonsurvivors with modern data. We cannot directly compare ages of attainment for menarche in the clinical literature with the average age of skeletons who have reached menarche in our skeletons. Skeletal samples of “known‐puberty” stage adolescents do not exist, meaning we cannot directly test our methods, and we are forced to use modern data to trace the tempo and synchronicity of the growth spurt. Even when comparing one archeological sample with another, hidden heterogeneity can hamper direct, simplistic or overly straightforward interpretations. Some measures human biologists use to understand the impact of environmental adversity on adolescent health are invisible to us, for example while a slowed pace of growth is seen as the best measure of nutritional stress in modern adolescents, our data is cross‐sectional meaning we cannot measure individual growth velocity. The subtle hormonal changes that signal the onset of puberty may have a limited effect on skeletal and dental tissues, and our current indicators of “onset” (canine root development, hook of the hamate bone) may not be sensitive enough for us to be able to compare age of onset in one skeletal group compared with another. Finally, small sample sizes due to a lower risk of mortality for adolescents, and the need for good preservation for sex and puberty stage estimations means we often have to temper our interpretations.

Working within these limitations, our understanding the health, growth, and development of this unique group has to potential to bridge the gap between human biology, historical sources, evolutionary and life history theory, and adaptive plasticity. Adolescent development reflects experiences from the womb into adulthood, and may resonate across generations. In sum, the future potential of adolescent bioarcheology is an exciting one.

## AUTHOR CONTRIBUTIONS

**Mary E. Lewis:** Conceptualization (lead); investigation (lead); writing – original draft (lead); writing – review and editing (lead).

## CONFLICT OF INTEREST

The author declares no conflict of interest.

## Data Availability

Data availability is not relevant to this paper as no new data was created for this research.
